# The Effect of Technical Training Provided by Agricultural Cooperatives on Farmers’ Adoption of Organic Fertilizers in China: Based on the Mediation Role of Ability and Perception

**DOI:** 10.3390/ijerph192114277

**Published:** 2022-11-01

**Authors:** Yuying Liu, Kaiyao Shi, Ziqi Liu, Ling Qiu, Yan Wang, Hao Liu, Xinhong Fu

**Affiliations:** College of Management, Sichuan Agricultural University, Chengdu 611130, China

**Keywords:** agricultural cooperatives, technical training, organic fertilizers, adoption, perception, ability, mediation effect, citrus planting, China

## Abstract

Organic fertilizers can be crucial in promoting sustainable agricultural development, but they are not used in a wide-ranging way among smallholder farmers in many developing countries. In China, cooperatives are considered essential subjects of agricultural technical training, but it is more common to join cooperatives without participating in their technical training. Thus, joining cooperatives or not cannot simply be used to assess the role of cooperatives in influencing the farmers’ production behavior. Based on survey data of 1160 citrus farmers in Sichuan Province, China, this study estimated the effect of the technical training provided by agricultural cooperatives on farmers’ adoption of organic fertilizers, taking into account the farmers’ ability and perception as the mediation variables. The findings showed that participating in the technical training provided by agricultural cooperatives could significantly enhance the likelihood that farmers will adopt organic fertilizers. The impact was 81.6% in influencing the farmer’s abilities and 7.64% in their perceptions of organic fertilizers. Furthermore, other variables, such as farm sizes, land transfers, and education levels, also make a difference in the effectiveness of the agricultural cooperatives’ technical training. This study provides support for developing pertinent policies to promote the complete adoption of agricultural cooperatives’ technical training functions and the widespread use of organic fertilizers.

## 1. Introduction

Chemical fertilizers have considerably increased grain production and hastened China’s agricultural development [1,2]. However, overuse of chemical fertilizers over the years has resulted in deteriorating soil quality, contaminated water, and increased greenhouse gas emissions [3,4]. The promotion of organic fertilizers is the main action the Chinese government has tried to conduct in order to decrease the usage of chemical fertilizers. (It is the “Action to Achieve Zero Growth of Pesticide Use by 2020” proposed by the Ministry of Agriculture and Rural Affairs of China in 2015. For more detailed information, please refer to: http://www.zzys.moa.gov.cn/gzdt/201503/t20150318_6309945.htm, (accessed on 18 March 2015). It is the “Action Program to replace chemical fertilizers with organic fertilizers for fruits, vegetables and tea” proposed by the Ministry of Agriculture and Rural Affairs of China in 2017. For more detailed information, please refer to: http://www.moa.gov.cn/nybgb/2017/derq/201712/t20171227_6130977.htm, (accessed on 27 December 2017). Compared with chemical fertilizers, organic fertilizers are more beneficial in mitigating climate change, preserving the soil’s fertility, and ensuring the excellence of agricultural products [3,5,6,7].

To some extent, adopting organic fertilizers can be considered adopting new technologies [8,9]. At this stage of development, adopting new technologies is the core of the growth of agriculture in China. It has been discovered that there are many similarities between the conclusions of domestic and international research on adopting new technology. We summarized the factors into three levels. Previous studies have shown that farmers’ adoption of new technologies depends on a variety of factors, such as individual features and household characteristics, which are influenced explicitly by household heads’ age, education levels, farm size, household size, farmers’ risk attitudes, personal preferences, technology perceptions, and membership in farm cooperatives [10,11,12,13,14,15]. At the same time, socioeconomic factors may also influence the adoption of organic fertilizers, such as policy environment, off-farm income, and the cost of adopting new agricultural technologies [16,17]. The technical factors include technical training, the ability to apply technology, and the new technology’s performance. Among them, farmers’ insufficient mastery of organic fertilizers technology and poor perceptions of organic fertilizers are crucial factors that hinder the adoption of organic fertilizers [18]. Due to farmers being limited by their abilities and perceptions, they often turn to external organizations for technical assistance. External technical assistance is provided to farmers mainly through technical training. There are usually three main types of technical training that farmers may participate in: technical training provided by government-assigned experts, fertilizers suppliers, and agricultural cooperatives.

Most of the technical training provided by the training subjects often has weak targeting, substantial limitations, a small service audience, and other defects. Through one-on-one training and field guidance, agricultural cooperatives have apparent advantages in training farmers and promoting agricultural technologies [19,20]. For example, regarding technology adoption, Wang et al. and Suvedi et al. found that farmers who join cooperatives have easier access to advanced technologies and make more rational technology-adoption decisions [21,22]. Regarding technology application, Kashiwagi [23] found that farmers with cooperative membership were more technically efficient and had more significant technological progress. In general, agricultural cooperatives have advantages in promoting agricultural technologies. Existing studies have analyzed the current situation of technology adoption mainly in terms of whether farmers have membership in cooperatives. However, not all farmers with cooperative membership are equally affected by agricultural cooperatives. For example, only farmers involved in agricultural cooperatives’ technical training are affected by the technical training function of cooperatives [24].

In reality, no more than 50% of farmers choose to adopt organic fertilizers in China [8,9,25,26], and an even smaller percentage could appropriately apply organic fertilizers [10,27]. Therefore, how can we improve the farmers’ adoption of organic fertilizers? According to the relevant information, the available research findings cannot answer whether agricultural cooperatives’ technical training can promote farmers’ adoption of organic fertilizers or explain the mechanism of action of technical training provided by agricultural cooperatives in influencing farmers’ adoption of organic fertilizers. Therefore, this study’s main goal is to give a more thorough evaluation of how the technical training provided by agricultural cooperatives affects farmers’ adoption of organic fertilizers. The focus on the mechanisms of technical training provided by agricultural cooperatives on farmers’ adoption of organic fertilizers will help deepen the understanding of the technology diffusion function of agricultural cooperatives and their role in promoting sustainable agricultural development. This study contributes to research on the use of organic fertilizers, the spread of agricultural technology, and the development of sustainable agriculture. First, we conduct an empirical analysis of how farmers’ adoption of organic fertilizers is affected by the technical training provided by agricultural cooperatives. According to earlier studies, farmers’ adoption of organic fertilizers is influenced by their membership in agricultural cooperatives. However, not all farmers who join cooperatives are equally influenced. Considering the function of technology diffusion of agricultural cooperatives, it is necessary to analyze further the effect of the technical training provided by agricultural cooperatives on farmers’ adoption of organic fertilizers. Second, we analyze the mediation role of farmers’ abilities and perceptions on the relationship between agricultural cooperatives’ technical training and farmers’ adoption of organic fertilizers. According to certain studies, farmers’ abilities and perceptions of new technology have a meaningful impact on the effectiveness of technology diffusion [13,17]. This study introduces two mediation variables: farmers’ ability with organic fertilizers and farmers’ perceptions of organic fertilizers. By estimating their mediation effects, we further explore the impact of technical training provided by agricultural cooperatives on farmers’ adoption of organic fertilizers in terms of ability and perception. Third, this study is in light of the cash crop, citrus. Most previous studies on promoting organic fertilizers technology are focused on grain crops, such as rice and soybeans. However, compared with traditional grain crops, citrus as a cash crop has a significant market profit and is more likely to cause the irrational use of fertilizers.

Other structures of this paper are as follows: In Section 2, we present the theoretical framework and hypotheses. Section 3 presents the model, data, and variable selection, while Section 4 presents the empirical results. Section 5 describes the conclusions and implications.

## 2. Theoretical Framework and Hypotheses

### 2.1. The Effect of the Technical Training Provided by Agricultural Cooperatives on Farmers’ Adoption of Organic Fertilizers

Several factors can influence whether or not new agricultural technologies are adopted by farmers [8,9]. According to earlier research, the following restrictions may prevent farmers from adopting organic fertilizers [21]. First, the lack of reliable agricultural extension agencies to promote organic fertilizers technology is the critical reason for the low proportion of organic fertilizers adoption in agriculture [28]. Technology diffusion theory requires adequate information spread to achieve technology diffusion [29]. However, farmers lack effective channels to learn more about organic fertilizers, reducing their willingness to adopt them. Second, farmers lack stable supply channels to obtain organic fertilizers. When farmers adopt organic fertilizers, they may lack sufficient financial support or efficient channels to purchase organic fertilizers [30]. Therefore, farmers will reduce or even avoid the adoption of organic fertilizers. Finally, the uncertainty of nutrient content and difficulties in using organic fertilizers technology may also hinder the farmers’ adoption of organic fertilizers [8]. On the one hand, farmers are worried that adopting organic fertilizers will increase their costs, resulting in higher selling prices and less competitiveness in the market for their farm produce. On the other hand, considering the uncertainty of organic fertilizers adoption, farmers will choose to continue to maintain their existing fertilization program based on experience due to risk aversion [31].

As an essential market participant in the supply of agricultural technologies, agricultural cooperatives have some advantages in promoting new agricultural technology. First, due to the closeness of agricultural cooperatives to farmers, they are often in a similar ecological environment as farmers [24]. On the one hand, agricultural cooperatives better understand the local climate and environmental conditions and can more accurately determine the suitable methods for organic fertilizers adoption. On the other hand, the language employed in technical training by agricultural cooperatives can be more in line with the cognitive characteristics of nearby farmers. Compared with technical training provided by other subjects, agricultural cooperatives as training sources can offer more forms of technical training [32], which can be more targeted to solve farmers’ problems in the production process. Therefore, the particular advantages of agricultural cooperatives are that they are better able to facilitate farmers’ adoption of new agricultural technologies [33]. Second, agricultural cooperatives can broaden farmers’ fertilizers supply channels. By purchasing in quantity, agricultural cooperatives can provide farmers with a continuous supply and stable, quality organic fertilizers [34]. Finally, new agricultural technology needs to be competitive in terms of price if it is to be popularized and adopted quickly. The agricultural cooperatives can promote the farmers’ rational adoption of organic fertilizers to boost the economic benefits of crop yields by reducing the number of chemical fertilizers applied while increasing the yield. As a result, farmers can sell their produce a higher price, further increasing their profitability. They will be more inclined to adopt organic fertilizers willingly [11,35].

Therefore, agricultural cooperatives’ technical training can promote farmers to adopt organic fertilizers. On the basis of the study done above, Hypothesis 1 was proposed:

**H1.**  *Technical training provided by agricultural cooperatives has a statistically significant, positive effect on farmers’ adoption of organic fertilizers.*

### 2.2. The Mediation Effect of Farmers’ Abilities of Technology between Technical Training Provided by Agricultural Cooperatives and Farmers’ Adoption of Organic Fertilizers

In production, inefficient technology and weak ability of technology are the main barriers to farmers’ technology adoption [36,37]. Agricultural cooperatives’ technical training can provide farmers with more knowledge about organic fertilizers and, through a variety of training forms, such as field guidance and one-to-one training, give farmers better abilities in organic fertilizers technology. On the one hand, the better abilities of organic fertilizers technology mean they are more likely to apply it to their agricultural production. Therefore, the level of farmers’ willingness to adopt organic fertilizers increased and can further improve the efficiency of crop yields. On the other hand, existing studies have found that most farmers prefer to use the agricultural technology that they are used to using. Farmers have better abilities with organic fertilizers technology, which means they are more familiar with it [38]. As a result, they are more willing to choose to use organic fertilizers. According to the theory of peer effect [13], farmers can enhance their abilities with organic fertilizers technology by interacting with farmers in the same cooperative, encouraging them to adopt organic fertilizers. Based on the above analysis, Hypothesis 2 was proposed:

**H2.**  *Farmers’ abilities with organic fertilizers technology mediate the relationship between agricultural cooperatives’ technical training and farmers’ adoption of organic fertilizers.*

### 2.3. The Mediation Effect of Farmers’ Perceptions of Technology between Technical Training Provided by Agricultural Cooperatives and Farmers’ Adoption of Organic Fertilizers

Technical training is vital for farmers to acquire knowledge about new technologies. From the agricultural cooperatives’ technical training, farmers can learn more about organic fertilizers and gain more profound perceptions of the value of organic fertilizers, which will influence their decisions on organic fertilizers adoption [39,40]. Due to distance proximity and the close relationship between farmers and agricultural cooperatives, farmers will gradually develop trust and dependence on agricultural cooperatives. Therefore, the technical training provided by agricultural cooperatives has a significant influence on the formation of the perceptions of the value of organic fertilizers than the technical training provided by other technical training subjects. 

The value of organic fertilizers can be separated into several dimensions, and we mainly select the economic and ecological significance of organic fertilizers. For the monetary value of organic fertilizers, first, given that Chinese farmers use excessive amounts of chemical fertilizers, the promotion of organic fertilizers technology enables farmers to make more rational fertilizers-adoption decisions and helps them to improve their yields. Therefore, considering the economic interest, farmers will adopt organic fertilizers. Second, farmers find it challenging to gather market information due to the information asymmetry in the market for agricultural products. Farmers can acquire more market information about their agricultural products from the agricultural cooperatives’ technical training, eliminating their disadvantages in terms of information access. Third, in the background of the growing popularity of the green food concept, more and more consumers are aware of their responsibility for environmental protection, resulting in greater participation in environmentally friendly behaviors, including the purchase of green agricultural products [41,42]. Moreover, the agricultural products produced by organic fertilizers technology are defined as green and can have a higher selling price in the agricultural product market. As a result, farmers are more likely to adopt green production techniques if they know more about the current situation of the farm product market.

For the ecological value of organic fertilizers, the technical training provided by agricultural cooperatives mainly affects farmers’ perceptions of organic fertilizers through two aspects: maintaining soil fertility and protecting the ecological environment. First, farmers discovered that organic fertilizers may improve soil structure and maintain soil fertility to provide a better environment for plants by taking part in the technical training provided by agricultural cooperatives [8,43,44]. At the same time, improving soil structure can protect crop growth from production risks and may motivate some farmers to adopt organic fertilizers [45]. Second, to some extent, the increasing scale of agricultural production has raised significant environmental problems [46]. In the context of increasing ecological degradation, adopting organic fertilizers can protect the ecological environment and reduce environmental issues [47]. During the technical training, farmers are led by agricultural cooperatives to preserve the ecological environment and realize that a perfect environmental setting benefits their and their families’ health. Farmers develop new perceptions of organic fertilizers’ human health and environmental benefits after participating in technical training provided by agricultural cooperatives. Thus, through technical training provided by agricultural cooperatives, farmers can realize the ecological value of organic fertilizers and become more willing to adopt them.

Based on the above analysis, Hypothesis 3 was proposed to mediate farmers’ perceptions of the value of organic fertilizers. Meanwhile, considering that organic fertilizers value has multiple dimensions, hypotheses 3a and 3b were proposed to mediate farmers’ perception of organic fertilizers’ economic value and ecological value.

**H3.**  *Farmers’ perceptions of the value of organic fertilizers mediate the relationship between technical training provided by agricultural cooperatives and farmers’ adoption of organic fertilizers.*

**H3a.**  *Farmers’ perceptions of the economic value of organic fertilizers mediate the relationship between technical training provided by agricultural cooperatives and farmers’ adoption of organic fertilizers.*

**H3b.**  *Farmers’ perceptions of the ecological value of organic fertilizers mediate the relationship between the technical training provided by agricultural cooperatives and farmers’ adoption of organic fertilizers.*

**Figure 1:**
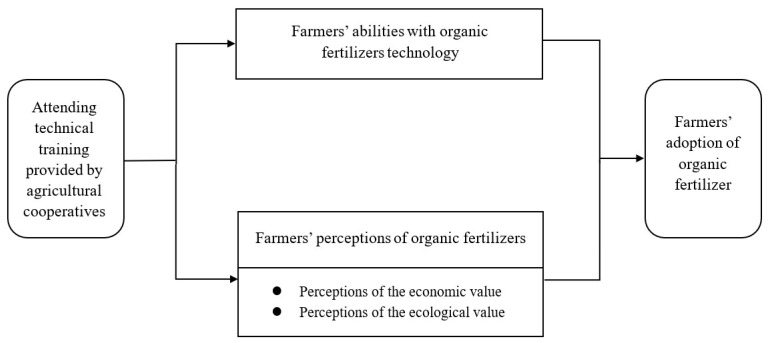
Theoretical model.

Figure 1 shows the theoretical model of this study.

## 3. Model, Data, and Variables

### 3.1. Model Specification

#### 3.1.1. Benchmark Model

This study’s primary goal is to estimate how much the technical training provided by agricultural cooperatives affects farmers’ adoption of organic fertilizers. According to earlier studies, a Probit model was used to determine the factors influencing farmers’ adoption of organic fertilizers. The Probit model is a regression model that may model dichotomous or binary outcome variables. The Probit model is a kind of regression model used to model dichotomous or binary outcome variables. The dependent variable, farmers’ adoption of organic fertilizers, takes binary values: 1 if a farmer adopted commercial organic fertilizers during the citrus planting last year, and 0 for otherwise. Since the dependent variable is binary, a Probit model was chosen as a benchmark for estimate.

(1) $$P(Y_{i}=1|Training_{i})=\beta_{0}+\beta_{1}Training_{i}+\beta_{2}Control_{i}+\beta_{3}Z_{i}+\mu_{3}\;$$

where $Y_{i}$ represents farmers’ adoption of organic fertilizers. $Training_{i}$ is an observed binary outcome variable, indicating whether farmers participated in the technical training provided by agricultural cooperatives. $Control_{i}$ is a vector of individual and household characteristics (e.g., age, gender, education, farm size, household size, and so on); $Z_{i}$ represents the district variables; $\mu_{1}$, $\mu_{2}$, and $\mu_{3}$ are constant terms; and $\beta_{1}$, $\beta_{2}$, and $\beta_{3}\;$ are parameters to be estimated.

#### 3.1.2. The Control Function Method

Endogenous problems may be the main reason for biased estimates of the effect of the agricultural cooperatives’ technical training on farmers’ adoption of organic fertilizers. First, endogenous problems may be caused by missing variables affecting farmers’ adoption of organic fertilizers. However, we selected several variables from individual characteristics, household characteristics, and geographical location. Many factors influencing farmers’ adoption of organic fertilizers may not be considered, which will usually produce inconsistent and biased results. Second, technical training participation may have a causal relationship with adopting organic fertilizers. From the above discussion, farmers’ technical training participation may affect the adoption of organic fertilizers. In addition, farmers’ adoption of organic fertilizers is influenced when deciding whether to participate in technical training provided by agricultural cooperatives. This endogeneity issue will cause the Probit model to provide results that are inconsistent and biased. Thus, it is of great necessity to control such endogeneity problems.

The endogeneity problem was addressed using the instrumental variable and control function methods. Even though both methods produce estimates that are consistent, the control function method is a more efficient estimator [48]. Therefore, we estimated the effect of the technical training provided by agricultural cooperatives on farmers’ adoption of organic fertilizers using the control function method.

This study estimates the potential endogeneity of technical training provided by agricultural cooperatives using Wooldridge’s control function method [49]. There are two stages in the control function method. In the first stage, Equation (2) examines the impact of the instrumental variable and control variable on the technical training provided by agricultural cooperatives and obtains the residual $\sigma_{i}$ by prediction. Furthermore, Equation (2) was as follows:

(2) $$Training_{i}=\alpha Z_{i}+\sigma_{i}+\theta \;$$

where $Z_{i}$ represents the factors that influence the participation in the technical training provided by agricultural cooperatives; α is a parameter to be estimated; $\sigma_{i}$ represents the residual; θ is a constant term.

Adding the residual $\sigma_{i}$ to the second stage regression of Equation (3) estimates, and the Equation (3) was as follows:

(3) $$Y_{i}=\beta Training_{i}+\gamma Control_{i}+\mu_{i}+\sigma_{i}+\eta \;$$

where $Y_{i}$ represents farmers’ adoption of organic fertilizers; $Training_{i}$ represents the technical training provided by agricultural cooperatives; $Control_{i}$ means the factors that influence the farmers’ adoption of organic fertilizers; β and γ are parameters to be estimated; $\sigma_{i}$ is the residual; $\mu_{i}$ is random disturbance term; η is a constant term.

#### 3.1.3. The Mediation Effect Model

Using the mediation effect model created by Baron and Kenny [50], we tested the mediation effect of farmers’ abilities of organic fertilizers technology and farmers’ perceptions of organic fertilizers between the technical training provided by agricultural cooperatives and farmers’ adoption of organic fertilizers using the following three models.

(4) $$Y_{i}=c_{0}+c_{1}Training_{i}+c_{2}Control_{i}+\varepsilon_{1}\;$$

(5) $$M_{i}=a_{0}+a_{1}Training_{i}+a_{2}Control_{i}+\varepsilon_{2}$$

(6) $$Y_{i}=b_{0}+c_{1}{}^{’}Training_{i}+b_{1}M_{i}+b_{2}Control_{i}+\varepsilon_{3}$$

where M represents mediation variables, including farmers’ abilities of organic fertilizers technology and farmers’ perceptions of organic fertilizers; Y represents the dependent variable—farmers’ adoption of organic fertilizers; and Training represents independent variables—the technical training provided by agricultural cooperatives. Equation (4) examines the relationship between Training and Y. Equation (5) investigates the link between Training and M. According to [50], the mediation-effect test procedure consists of the following four steps. First, that both $c_{1}$ and $a_{1}$ are significant is a prerequisite for continuing the estimation. When $c_{1}$ and $a_{1}$ are substantial, Equation (6) is estimated to test whether Training and M are dependent on Y. Second, when both $a_{1}$ and $b_{1}$ are statistically significant, it can be confirmed that M mediates the relationship between Training and Y. Otherwise, go directly to the last step. Third, when the coefficients $c_{1}{}^{’}$ and $c_{1}$ are statistically significant, and $c_{1}{}^{’}<c_{1}$, a partial mediating effect exists. When $c_{1}{}^{’}$ is not significant, it has a full mediating effect. Fourth, the Sobel test is carried out. Meanwhile, the value of the mediating effect is $a_{1}b_{1}$, and $a_{1}b_{1}/c_{1}$ is the proportion of the mediating development in the product.

#### 3.1.4. Heterogeneous Test

According to previous studies [11,33,51,52,53], individual characteristics, household characteristics, and geographical location are indispensable factors affecting farmer’s adoption of organic fertilizers. To further understand the agricultural cooperatives to provide technical training to the influence of different farmer groups, we also studied the agricultural cooperatives to provide technical training for different farm scales, land transfers, education levels, and the influence of the economic region of farmers using organic fertilizer. The farmers were divided into two groups according to different influencing factors. And the regression was conducted to observe the differences between the two groups of farmers. At last, we drew conclusions based on the results.

### 3.2. Data

#### 3.2.1. Study Area

Citrus is a fruit that is domesticated worldwide. Whether the citrus-planting process is green affects the health of growers and the production environment widely [24]. Both the citrus-planting area and its output in China rank first in the world, and the output is over 51 million tons, with 2.7 million hectares of planting area in 2020 [54]. The citrus industry is a foundation of the economy in Sichuan, particularly in the steep and hilly regions. Furthermore, the planting area of citrus was over 310,000 hectares, and the output was more than 4.8 million tons, ranking fourth in China [24]. Meanwhile, considering in the upper reaches of the Yangtze River in Sichuan, farmers’ usage of organic fertilizers in Sichuan, especially in mountainous and hilly areas, is not only good for the local environment but also suitable for the middle and lower reaches of the Yangtze River [24]. Thus, citrus planting farmers in Sichuan Province were selected to be the research object.

#### 3.2.2. Sampling Procedure

**Figure 2:**
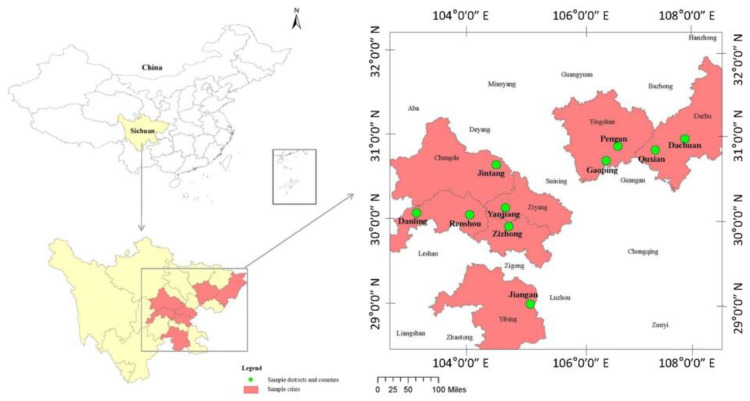
Distribution of sample counties.

In this study, we used the data collected during a field survey carried out in southwest China’s Sichuan Province in August 2020 and August 2021. For selecting samples of the household to support the empirical analysis of this study, we employed a multistage sampling procedure. After choosing the three major economic zones on purpose, ten counties were carefully selected. In this process, a stratified sampling method was used based on the distribution of citrus yield and the overall number of agricultural cooperatives specializing in citrus production and marketing. These include Renshou, Danling, Renshou, and Yanjiang in the Chengdu Plain economic zone; Zizhong and Jiangan in the south Sichuan economic zone; and Jintang, Gaoping, Pengan, Dachuan, and Qu in the northeast Sichuan economic zone. In the next stage, based on the annual citrus production, two to four villages were stratified, sampling from each of the fourteen counties, and random selection of one to four agricultural cooperatives from each village was performed (See Figure 2). 

#### 3.2.3. Instrument 

The data were collected using a structured questionnaire with a face-to-face survey approach. The questionnaire included individual and household characteristics (e.g., gender, age, education, health, farm size, household size, land transfer, and non-farm workers), perceptions of organic fertilizers, participation in technical training, and so on. In total, 1160 valid questionnaires were collected.

### 3.3. Variable Selection and Descriptive Statistics

#### 3.3.1. Variable Selection

Dependent variable: Since we want to analyze the impact of technical training provided by agricultural cooperatives on farmers’ adoption of organic fertilizer, it is essential to evaluate farmers’ adoption of organic fertilizer. According to previous research [11,51,55], we set the dependent variable to whether farmers formally adopt organic fertilizer in citrus cultivation. Based on the statistical data, bagged commercial organic fertilizers are the type of fertilizers that farmers choose to apply with a higher probability because they are more easily absorbed and more effective [3,26]. Therefore, we chose bagged commercial organic fertilizers to represent organic fertilizers. The dependent variable gave the value of 1 if a farmer adopted commercial organic fertilizers during the citrus planting and was 0 otherwise.

Independent variable: Following previous studies [11,52], this study used a dummy variable to track whether or not farmers participated in the technical training provided by agricultural cooperatives to identify farmers’ training participation behaviors. Our independent variable gave the value of 1 if a farmer participated in the technical training provided by agricultural cooperatives before and 0 otherwise. In this research, technical training provided by agricultural cooperatives mainly focuses on the advantages and disadvantages of traditional chemical fertilizer and organic fertilizer, fertilizers’ application time, topdressing opportunity, application times, application amount, as well as the varied selection of commercial organic fertilizer, homemade organic fertilizer manufacture method, etc.

Mediation variables: The study’s second objective is to estimate the mediating effect of farmers’ ability and perception of technology on the relationship between the technical training provided by cooperatives and farmers’ adoption of organic fertilizers. Based on the previous analyses [55,56,57], we selected farmers’ abilities with organic fertilizers technology, perceptions of the economic importance of organic fertilizers, and perceptions of the ecological value of organic fertilizers as our mediation variables. The specific measures are shown in Table 1.

Control variables: Based on previous studies [3,10,11,33,53], we supposed that variables that may have influenced the farmers’ adoption of organic fertilizers mainly include individual and household characteristics. Specifically, the unique features include the household head’s gender, age, education levels, and health. The household characteristics include farm size, home size, the number of non-farm workers in the family, and the experience of land transfer.

District variables: The geographical location is an indispensable factor affecting farmers’ adoption decisions. Therefore, the differences between different economic zones may affect the farmers’ adoption of organic fertilizers. The agricultural cooperatives in the sample came from three of the five major economic zones in Sichuan Province, namely the Chengdu Plain economic zone, the south Sichuan economic zone, and the northeast Sichuan economic zone.

#### 3.3.2. Descriptive Statistics

Table 1 presents the definitions of the variables used in this study and their summary statistics. Around 68% of respondents participated in the technical training provided by agricultural cooperatives, and about 78.9% of the respondents adopted organic fertilizers. Male respondents made up 67.6% of the total number; the average age of the respondent was 55.7 years, with average schooling of 7.48 years. Additionally, the average size of the citrus grove was 1.69 hectares. Just 33.7% of respondents had the experience of land transfer. The average household size and non-farm workers were 4.53 members and 1.3 members, respectively, which indicated that the sample home’s non-farm worker members made up 30.6% of the total number of household members. The respondents came from agricultural cooperatives in different economic zones, among which the proportion of farmers from agricultural cooperatives in the Chengdu Plain economic zone, the south Sichuan economic zone, and the northeast Sichuan economic zone were 53.7%, 22.8%, and 23.5%, respectively.

Table 2 presents the sample mean values between participants and nonparticipants of the technical training provided by agricultural cooperatives, along with the mean differences between the two groups. At first glance, farmers who participated in the technical training provided by agricultural cooperatives were 37.5% more likely to adopt organic fertilizers. The technical training variable positively and significantly impacted farmers’ adoption of organic fertilizers at a 1% level. In particular, relative to nonparticipants, participants were more educated, had larger farms, and were more likely to be female, while they had less experience with land transfer and were younger and in better health. However, because these comparisons are just descriptive and do not take confounding factors into account, the observations are inconclusive.

## 4. Empirical Estimation Results

### 4.1. Benchmark Model Results

To study the relationship between farmers’ adoption of organic fertilizers and the technical training provided by agricultural cooperatives, we built three Probit models in Section 3.1 to make the base estimate.

Table 3 shows the Probit-model-estimated results, and the variables influencing farmers’ adoption of organic fertilizers are reported in this base estimate. Given that the explanatory variable coefficients are not directly explained, we computed and present the estimated marginal effects results. According to columns two and three of Table 3, the following results of model 1 were obtained. With a marginal effect of 0.298, the technical training variable positively and significantly impacted farmers’ adoption of organic fertilizers behavior at a 1% level. After adding control variables to model 2, the marginal effect was 0.259, which was positive and statistically significant at the 1% level. After adding control and district variables to model 3, the marginal effect was 0.264, which was positive and statistically significant at the 1% level. According to this marginal effect, farmers who took part in technical training provided by agricultural cooperatives are 26.4% more likely to use organic fertilizer than those who did not, which is consistent with the findings of Liu et al. [24]. This result shows that technical training provided by agricultural cooperatives can significantly increase the propensity of farmers to adopt organic fertilizers. There are several reasons for this. First, the lack of effective channels to learn about new technologies is why farmers do not adopt them. Technical training provided by cooperatives can spread new technology and teach farmers how to use organic fertilizers correctly [23,58]. Second, agricultural cooperatives can be essential in accelerating the adoption of agricultural technologies. According to previous studies, the technical training farmers receive and the influences of other farmers in agricultural cooperatives impact farmers’ behavior [59]. Farmers may spread the knowledge they learn to others and influence other farmers’ adoption of new technology due to the high potential peer effects among farmers who grow the same crops [13]. Third, unlike the technical training provided by other subjects, technical training provided by agricultural cooperatives not only provides traditional one-time training but also includes in-field guidance [32]. Thus, Hypothesis 1 (H1) was confirmed.

### 4.2. Endogeneity Checks

Following the analysis of Section 3.1.2, there are potential endogeneity issues associated with the technical training participation variable. The instrumental variable and control function methods were suggested as solutions to the endogeneity problem. Additionally, it is necessary to select an appropriate instrumental variable. According to the theory of peer effects [60], the behavior decisions of farmers in the same village will influence each other. This kind of mutual influence is more common in rural China, where people are familiar with each other [13] Furthermore, based on previous studies [24,61], we selected the instrumental variables using the mean value of the times of the technical training participation that farmers in same village, excluding those interviewed (from now on referred to as “ratio of training”). Suppose that $V_{training}=\left(training_{1}+training_{2}+\cdots +training_{n-1}\right)/\left(n-1\right)$, where n represents the whole sample size of farmers included in this study. The technological surroundings and access to information may affect the possibility of adopting agricultural technologies [15,55]. In detail, the more farmers in a village participate in the technical training provided by agricultural cooperatives, the probability of other farmers in the village participating in the training provided by agricultural cooperatives will increase. At the same time, the farmer’s organic fertilizer adoption will not be affected by the ratio of other villagers participating in the agricultural cooperative training. Additionally, the estimation results demonstrate that the relationship between the ratio of training and the technical training participation choices is statistically significant at the 1% level and passes the over-identification test. Meanwhile, the ratio of training and the adoption of organic fertilizers, however, do not have a statistically meaningful relationship. It shows that the instrumental variable of “the ratio of training” is plausible. The results of using the control function method (CFA) are presented in Table 4.

Table 4 reports the results of the CFA model. Farmers who took part in the technical training provided by agricultural cooperatives were 53.9% more likely to adopt organic fertilizers than non-participants. This indicated that the effects of the agricultural cooperative’s technical training are underestimated in the Probit model. The above conclusion demonstrates that endogeneity checks are necessary.

### 4.3. Robustness Checks

In this part, the robustness of the results is examined in two ways to verify the above results: (1) To further check the robustness of the results of the benchmark model, we used the OLS model to estimate. (2) To further check the robustness of the results of endogeneity checks, we used the IV-Probit model to estimate.

#### 4.3.1. Change Benchmark Model

Table 4 also reports the results of changing the benchmark model, estimated by the OLS model [51]. The causal effect of participating in the technical training provided by agricultural cooperatives on farmers’ adoption of organic fertilizers was 0.338. At the 1% level, the coefficient is statistically significant. This finding shows that the benchmark model’s results are robust considering they are consistent with the results of the Probit model.

#### 4.3.2. Change the Model of Endogeneity Checks

Table 5 reports the results of changing the model of endogeneity checks, estimated by the IV-Probit model. In column three, the results of the first stage show that the IV increased farmers’ adoption of organic fertilizers at a 1% level. As can be seen from column four, the IV-Probit model training variable’s coefficient was 0.790, which is more significant than that in the Probit model, which shows that the effects of the agricultural cooperatives’ technical training are underestimated in the Probit model. The participation in agricultural cooperatives’ technical training being treated as an exogenous variable in the regression of the Probit model may be the main reason.

### 4.4. Mediating Effect

#### 4.4.1. Mediating Effect of Farmers’ Abilities of Organic Fertilizers Technology

Table 6 shows the mediating effect of farmers’ abilities with organic fertilizers technology. Based on the model specification in Section 3.1, firstly, we tested the relationship between the technical training provided by agricultural cooperatives and the farmers’ abilities with organic fertilizers technology. Then, we estimated the effects of the participation in the agricultural cooperatives’ technical training and farmers’ abilities with organic fertilizers technology on farmers’ adoption of organic fertilizers. In Table 6, we present the results.

The coefficient of the agricultural cooperatives’ technical training in column three was significantly positive. The coefficient of farmers’ abilities of organic fertilizers technology in column four was insignificant. These indicate that farmers’ abilities of organic fertilizers technology play a fully mediating role in the relationship between the agricultural cooperatives’ technical training and the farmers’ adoption of organic fertilizers. Mastering a technology increases farmers’ confidence in applying this technology to production practice, increasing the probability of farmers’ technology adoption. The results showed that the technical training provided by agricultural cooperatives, mainly via the degree of farmers’ abilities with organic fertilizers technology, affects farmers’ adoption of organic fertilizers. Thus, Hypothesis 2 (H2) was supported.

#### 4.4.2. Mediating Effect of Farmers’ Perceptions of Organic Fertilizers

Table 7 shows the mediation role of farmers’ perception of organic fertilizer. According to Section 3.1 of the model specification, we first tested the relationship between the technical training provided by agricultural cooperatives and the farmers’ perceptions of organic fertilizers. After that, we evaluated the influence of farmers’ participation in technical training provided by agricultural cooperatives and their views on organic fertilizers on farmers’ adoption. The results as shown in Table 8.

As shown in columns three and four in Table 7, farmers’ adoption of organic fertilizers is greatly influenced by the technical training provided by agricultural cooperatives. The coefficient of farmers’ perceptions of the economic value of organic fertilizers in column five was also significant. This means that in the relationship between technical training provided by agricultural cooperatives and farmers’ adoption of organic fertilizers, farmers’ perception of the economic value of organic fertilizers plays a partial intermediary role. Through further calculation, this paper found that the intermediary effect of economic value perception of organic fertilizers was 0.293, accounting for 5.1% of the total effect. In other words, technical training provided by agricultural cooperatives has a 5.1% impact on farmers’ adoption of organic fertilizers through farmers’ perception of the economic value of organic fertilizers. Thus, Hypothesis 3a (H3a) was verified.

Meanwhile, in Table 8, the factors of agricultural cooperatives’ supply technical training and farmers’ perceptions of the ecological value of organic fertilizers were not significant in column five. Furthermore, we performed a Sobel test to evaluate the mediating effects of farmers’ perceptions of the ecological value of organic fertilizers. The Sobel test results indicated that the mediator effect of farmers’ perceptions of the environmental value of organic fertilizers was statistically significant at 5%. Through further calculation, the mediator effect of farmers’ perceptions of the ecological value of organic fertilizers was 0.479, occupying 2.54% of the total effect. In other words, agricultural cooperatives provide technical training for farmers on the impact of using organic fertilizer; 2.54% is produced by farmers’ perception of ecological value of organic fertilizers. Thus, Hypothesis 3b (H3b) was supported.

### 4.5. Heterogeneous Impact

The results presented in Table 9 and Table 10 generally show that even among other groups of farmers, the technical training provided by agricultural cooperatives tends to have a positive and significant impact on the farmers’ adoption of organic fertilizers at the level of 1%.

#### 4.5.1. Disaggregated Effect by Farm Size

Our samples come from the citrus area in Sichuan, which is mainly mountainous and hilly terrain. As a result, the average size of the citrus grove was smaller than the citrus areas in other provinces. The technical training variable’s coefficients for the larger and smaller farm groups were 0.168 and 0.276, respectively, according to the second and third columns of Table 9. The coefficients for different farm groups were all positive and statistically significant, and smaller farms can benefit from the technical training provided by agricultural cooperatives for farmers using organic fertilizer. According to survey data, one possible reason is that adopting organic fertilizers was more common among farmers with larger farms. Hence, the technical training provided by agricultural cooperatives had a more significant impact on adopting organic fertilizers by smaller farmers than by larger farmers. That discovery is consistent with Xiang et al. and Mao et al. for China and Velayudhan et al. for India, demonstrating that farmers are more likely to adopt organic fertilizers if their farms are smaller [5,51,62].

#### 4.5.2. Disaggregated Effect by Land Transfer

As shown in columns four and five of Table 9, for the land-conversion experience group and no land-conversion experience group, the coefficients of technical training variable were 0.304 and 0.245, respectively. The coefficients for both land-transfer groups were positive and statistically significant. The impact of technical training provided by agricultural cooperatives on farmers’ adoption of organic fertilizers can be enhanced through land-transfer experience. The findings suggest that, with the land transfer, farmers appear to expand the size of citrus-planting orchards [25]. As farming expands, the likelihood of farmers participating in the technical training provided by agricultural cooperatives to adopt new technology is higher. Hence, the technical training provided by agricultural cooperatives had a more significant impact on the adoption of organic fertilizers by farmers with land-transfer experience than by farmers without land-transfer experience.

#### 4.5.3. Disaggregated Effect by Educational Level

According to the findings, which are shown in columns six and seven of Table 9, the technical training variable’s coefficients were 0.331 and 0.193 in the groups with lower levels of education (≤7 years) and higher levels of education (>7 years), respectively. Both of the coefficients in the different education-level groups were positive and statistically significant, and lower education can enhance the positive effect of the technical training provided by agricultural cooperatives on farmers’ adoption of organic fertilizers. A possible explanation is that farmers with more education usually had more confidence in their experience. Their willingness to participate in technical training provided by agricultural cooperatives is not strong. Hence, the technical training provided by agricultural cooperatives had less impact on organic fertilizer adoption by farmers with higher education than by those with lower education. This finding is similar to Pan et al., who showed that farmers are more inclined to adopt organic fertilizers the less educated they are [32].

#### 4.5.4. Disaggregated Effect by Economic Zone

Columns two, three, and four in Table 10 show the variable coefficients of technical training of the groups located in the Chengdu Plain economic zone, the south Sichuan economic zone, and the northeast Sichuan economic zone were 0.165, 0.328, and 0.390, respectively. In the south Sichuan economic zone and northeast Sichuan economic zone, the coefficients of different economic zones were all positive, reaching a statistically significant level of 1%, indicating that it can enhance the positive impact of technical training provided by agricultural cooperatives on farmers’ adoption of organic fertilizer. According to previous studies [33,53], the adoption of green technology is related to geographical location. A possible reason for this is that agricultural product markets in the Chengdu Plain economic zone were more mature than others. Therefore, the more familiar groups in the Chengdu Plain economic zone are with green technology and agricultural products, and more likely it is that farmers will use organic fertilizer.

## 5. Conclusions and Implications

### 5.1. Conclusions

Farmers’ inability to use new technology is mainly impacted by their lack of knowledge and skill. Therefore, this study made a core analysis of the impact of technical training provided by agricultural cooperatives on farmers’ use of organic fertilizer and discussed its influencing mechanism from the perspective of farmers’ technical abilities and perceptions. By taking citrus growers in China as samples, this study has better understood the influence of technical training on adopting organic fertilizers by farmers in agricultural cooperatives, thus contributing to the literature.

Some conclusions were drawn through theoretical analysis and empirical estimation. First, farmers’ adoption of organic fertilizers can be promoted through the technical training provided by agricultural cooperatives. Second, farmers’ abilities of organic fertilizers technology, farmers’ perceptions of the economic value of organic fertilizers, and farmers’ perceptions of the ecological value of organic fertilizers play a mediating role in the technical training provided by agricultural cooperatives to promote farmers’ adoption of organic fertilizers. Specifically, farmers’ abilities with organic fertilizers technology (81.56%) play a fully mediating role, while farmers’ perceptions of the economic value of organic fertilizers (5.1%) and farmers’ perceptions of the ecological value of organic fertilizers (2.54%) play a partially mediating role. Third, smaller farm sizes, farmers with land-transfer experience, farmers with lower education, and agricultural cooperatives in the south Sichuan economic zone and northeast Sichuan economic zone positively influence farmers’ adoption of organic fertilizers.

The fact that we only paid attention to citrus farmers in China and ignored other crop species is a limitation of this study. Further research including different crops and different regions is required to examine the external validity of our findings given the growing significance of technical training provided by agricultural cooperatives in enhancing the likelihood that farmers will adopt organic fertilizers. Moreover, this might contribute to a better understanding of how technical training affects the adoption of agricultural technology. Future research could focus on other crucial questions, such as comparing the difference in training effects of various training organizations that use the same training form. 

### 5.2. Implications

The conclusions of this study have significant implications for encouraging Chinese farmers to adopt organic fertilizers. First, the conclusion further supports that the government should adopt incentives, such as subsidies, for participating in the technical training provided by agricultural cooperatives and introduce additional policies to improve the system of technical training provided by agricultural cooperatives. Thus, farmers perceive the authority of the technical training provided by agricultural cooperatives, so they may voluntarily join the technical training provided by agricultural cooperatives. Second, agricultural cooperatives’ technical training influenced farmers’ adoption of organic fertilizers by affecting their ability and perception. When agricultural cooperatives are the subject of technical training, they should focus on farmers’ abilities of organic fertilizers technology and enhance their perceptions of organic fertilizers. Through demonstration cases, technical explanations, and other forms, farmers can feel the benefits of adopting organic fertilizers and thus choose to adopt organic fertilizers. Third, different farm sizes, land transfers, farmers’ education levels, and economic zones make a difference in the effectiveness of the technical training provided by agricultural cooperatives. Thus, agricultural cooperatives should take complete account of the differences among farmers and adopt different approaches to their technical training. For example, agricultural cooperatives should consider theoretical technical training in organic fertilizers for farmers who lack basic knowledge of organic fertilizers. For farmers with a basic understanding of organic fertilizers, agricultural cooperatives should consider field technical training in organic fertilizers, such as field instruction and other forms, to make technical training more accessible and relevant for different farmers.

## Figures and Tables

**Table 1:** The definitions and summary statistics of the variables used in the analysis.

Variables		Definition	Mean	SD
Dependent variable				
Organic fertilizers adoption		1 if a farmer adopted commercial organic fertilizers last year, 0 otherwise	0.789	0.408
Independent variable				
Technical training		1 if a farmer participated in the technical training provided by agricultural cooperatives, 0 otherwise	0.680	0.467
Mediation variables				
Abilities of organic fertilizers technology		1 if a farmer mastered organic fertilizers technology, 0 otherwise	0.657	0.475
Perceptions of the economic value of organic fertilizers		To what extent a member knows about the economic value of organic fertilizers (from 1 = almost no idea to 5 = perfectly understanding)	3.825	0.930
Perceptions of the ecological value of organic fertilizers		To what extent a member knows about the ecological value of organic fertilizers (from 1 = almost no idea to 5 = perfectly understanding)	2.066	1.285
Control variables				
Individual characteristics	Gender	1 if the household head is male, 0 otherwise	0.720	0.449
	Age	Age of the household head (years)	55.720	9.526
	Education levels	Formal education of the household head (years)	7.480	3.522
	Health	The household head is very healthy (1 = strongly disagree; 2 = disagree; 3 = general; 4 = agree; and 5 = strongly agree)	4.065	0.793
	Farm size	The total size of citrus-planting orchards (hectares)	1.695	4.936
Household characteristics	Land transfer	1 if a farmer had the experience of land transfer, 0 otherwise	0.337	0.473
	Non-farm workers	Number of household members who are non-farm workers	1.296	1.063
	Household size	Number of household members who share meals	4.534	1.603
District variables				
Chengdu Plain economic zone		1 if the sample is located in Chengdu Plain economic zone, 0 otherwise	0.537	0.499
South Sichuan economic zone		1 if the sample is located in south Sichuan economic zone, 0 otherwise	0.228	0.419
Northeast Sichuan economic zone		1 if the sample is located in northeast Sichuan economic zone, 0 otherwise	0.235	0.424

Note: SD, standard deviation.

**Table 2:** Mean difference in characteristics between participants and nonparticipants of the technical training provided by agricultural cooperatives.

Variables	Nonparticipants	Participants	Diff
Organic fertilizers adoption	0.534 (0. 026)	0.909 (0.010)	0.375 ***
Gender	0.752 (0.022)	0.705 (0.016)	−0.047 *
Age	58.809 (0.491)	54.265 (0.328)	−4.544 ***
Education	6.267 (0.192)	8.051 (0.117)	1.784 ***
Health	3.930 (0.044)	4.128 (0.027)	−0.198 ***
Farm size	16.569 (2.973)	29.583 (2.863)	13.014 ***
Land transfer	0.375 (0.025)	0.319 (0.017)	−0.055 *
Non-farm workers	1.313 (0.058)	1.288 (0.037)	−0.025
Household size	4.472 (0.088)	4.563 (0.055)	0.091
Chengdu Plain economic zone	0.520 (0.026)	0.545 (0.018)	0.025
South Sichuan economic zone	0.221 (0.022)	0.231 (0.015)	−0.010
Northeast Sichuan economic zone	0.259 (0.023)	0.224 (0.015)	−0.034

Note: Standard errors are reported in parentheses; * and *** represent statistical significance at the 10% and 1% levels, respectively.

**Table 3:** The effects of the technical training provided by agricultural cooperatives on the farmers’ adoption of organic fertilizers: Probit model estimation.

Variables	Model 1	Model 2	Model 3
	Marginal Effects	Marginal Effects	Marginal Effects
Technical training	0.298 ***(0.017)	0.259 *** (0.018)	0.264 *** (0.018)
Gender		−0.028 (0.024)	−0.014 (0.024)
Age		0.019 ** (0.009)	0.020 ** (0.009)
Education		0.004 (0.004)	0.003 (0.004)
Health		0.034 ** (0.014)	0.040 *** (0.014)
Farm size		0.000 (0.000)	0.000 (0.000)
Land transfer		−0.034 (0.022)	−0.007 (0.023)
Non-farm workers		−0.004 (0.013)	−0.002 (0.013)
Household size		0.018 ** (0.009)	0.019 ** (0.009)
South Sichuan economic zone			−0.102 *** (0.027)
Northeast Sichuan economic zone			−0.141 *** (0.030)
Wald	191.11	218.02	219.23
Pseudo R2	0.1684	0.2093	0.2340

Notes: ** and *** represent statistical significance at the 5% and 1% levels, respectively. Standard errors are reported in parentheses.

**Table 4:** Estimates of the CFA model and OLS model.

Variables	CFA Model	OLS Model
Technical training	0.539 *** (0.114)	0.338 *** (0.028)
Control variables	Controlled	Controlled
District variables	Controlled	Controlled
Residual	0.493 *** (0.109)	
Constant	−0.469 *** (0.093)	−0.325 (0.273)
Chi-square	25.61	0.247

Notes: *** represents the statistical significance at 1%; robust standard errors are in parentheses.

**Table 5:** Estimates of the IV-Probit model.

Variables	Organic Fertilizers Adoption	Technical Training	Organic Fertilizers Adoption
Technical training	0.264 *** (0.018)		0.790 *** (0.066)
IV (Ratio of training)		0.603 *** (0.050)	
Control variables	Controlled	Controlled	Controlled
District variables	Controlled	Controlled	Controlled
Constant		0.136 (0.330)	

Notes: *** represents statistical significance at the 1% levels; robust standard errors are in parentheses.

**Table 6:** The results of the mediating effects of farmers’ abilities with organic fertilizers technology.

Variables	Organic Fertilizers Adoption	Abilities of Organic Fertilizers Technology	Organic Fertilizers Adoption
Technical training	0.264 *** (0.018)	0.957 *** (0.008)	0.122 (0.098)
Abilities of organic fertilizers technology			0.225 ** (0.096)
Control variables	Controlled	Controlled	Controlled
District variables	Controlled	Controlled	Controlled
R-squared		0.903	0.254

Note: *** and ** represent the statistical significance at 1% and 5% respectively; robust standard errors are in parentheses.

**Table 7:** Mediating effect results of farmers’ perception of organic fertilizer.

Variables	Organic Fertilizers Adoption	Perceptions of the Economic Value of Organic Fertilizers	Perceptions of the Ecological Value of Organic Fertilizers	Organic Fertilizers Adoption
Technical training	0.264 *** (0.018)	0.293 *** (0.078)	0.479 *** (0.080)	0.243 *** (0.018)
Perceptions of the economic value of organic fertilizers				0.04 6 *** (0.012)
Perceptions of the ecological value of organic fertilizers				0.014 (0.009)
Control variables	Controlled	Controlled	Controlled	Controlled
District variables	Controlled	Controlled	Controlled	Controlled

Notes: *** represents statistical significance at the 1% levels; robust standard errors are in parentheses.

**Table 8:** The results of the Sobel test.

Variables	Sobel Test
	Organic Fertilizers Adoption
Perceptions of ecological value of organic fertilizers	0.020 ** (0.009)
R-squared	0.250

Note: ** represent the statistical significance at 5%; robust standard errors are in parentheses.

**Table 9:** Effects disaggregated by groups’ farm sizes, land transfer, and education levels.

Variables	Farm Size		Land Transfer		Education Levels	
	Group with Larger Farm Size (>1.67 hectare)	Group with Smaller Farm Size (≤1.67 hectare)	Group Had Experience with Land Transfer	Group Had No Experience with Land Transfer	Lower-Educated Group (≤7 years)	Higher-Educated Group (>7 years)
Technical training	0.011 *** (0.004)	0.018 *** (0.001)	0.304 *** (0.032)	0.245 *** (0.021)	0.331 *** (0.024)	0.193 *** (0.025)
Control variables	Controlled	Controlled	Controlled	Controlled	Controlled	Controlled
District variables	Controlled	Controlled	Controlled	Controlled	Controlled	Controlled
Constant	0.162 (0.226)	−0.232 ** (0.101)	−2.634 (2.721)	−3.537 ** (1.453)	−4.035 (3.658)	−0.237 (1.585)
Pseudo R^2^	0.009	0.016	0.2386	0.2330	0.2829	0.1394
Observations	138	994	391	769	533	627

Note: The average farm size of the sample household is 1.67 hectare; the average education level of the sample household heads is seven years; *** and **, respectively, represent statistical significance of 1% and 5%; heteroskedasticity–robust standard error in parentheses.

**Table 10:** Effects disaggregated by groups’ economic zone.

Variables	Chengdu Plain Economic Zone	South Sichuan Economic Zone	Northeast Sichuan Economic Zone
Technical training	0.165 *** (0.025)	0.328 *** (0.035)	0.390 *** (0.027)
Control variables	Controlled	Controlled	Controlled
Constant	−3.127 ** (1.522)	−6.250 * (3.273)	4.449 (3.713)
Pseudo R^2^	0.1588	0.2557	0.3114
Observations	623	264	273

Note: ***, **, and * represent the statistical significance at 1%, 5%, and 10%, respectively; robust standard errors are in parentheses.

## Data Availability

The authors may provide raw data if necessary.
